# Mr. Hawker, on Epidemical Fever

**Published:** 1805-10-01

**Authors:** Peter Hawker

**Affiliations:** Tullamore


### To Dr. BRADLEY.

SIR,

IN perusing the 72d Number of the Medical Journal, I was much edified by the luminous History of the Spanish Epidemical Fever, given by the learned gentleman who signs himself W. Domeier, Physician to the Forces. But notwithstanding the classical elegance and perspicuity of style in which this paper is written; notwithstanding the acute ingenuity of research with which the physician to the forces has traced the causes of this fatal epidemic; and notwithstanding the profound judgment he has displayed in laying down his prophylaxis and plan of cure; there are some few passages which, to my dull comprehension, are not wholly intelligible; and there are some points of doctrine advanced, upon which, for want of sufficient information, perhaps, I still retain a degree of scepticism. The author commences by announcing the discovery of important truths, which he presumes will be attended with the fate of Cassandra's Prophecies, Gallileo's Discoveries, &c. He, however, comforts himself with the reflection, that the discussion of matters belonging to the practice of physic, until infallible cures shall be discovered, is always useful. From this observation it would seem, that the Doctor himself entertains some little doubts about the infallibility of his system (if system it may be called) notwithstanding the expressions of confidence which so frequently occur in his Essay; as, "This being proved." "The reader having been convinced by what has been said." "If any one doubts, &c."

The four first pages are taken up in informing his readers of what the generality of them have no doubt, that the human constitution is much influenced as to health or disease by the state of the atmosphere; this he considered as peculiarly modified in different countries, producing thereby diseases peculiar to each; hence, he denominates Egypt the land of ophthalmy; the Antilles of yaws; England of gout; America of syphilis; and the *North Sea*, the *land* of scurvy. "This being proved," says he, "let us now consider, &c."

What a pity it is, that "the limited room of your Journal" should have precluded this philosopher from explaining the peculiarities of atmosphere which induce syphilis, gout, &c. He then proceeds to state, that an atmosphere fit for the purposes of health and life, should contain twenty-seven parts oxygen, seventy nitrogen, and three carbonic acid gas; and pronounces, that any aberration from this standard is capable of producing disease or death. This is certainly a recent discovery, for it has generally been supposed that the fortuitious addition of carbonic acid air to the other component parts, was not absolutely necessary to the purposes of respiration. This preliminary discussion being concluded, he proceeds to open his subject, the epidemy of Spain; and here it is well that he has designed by a specific name the subject he intended to treat of; the Essay being as applicable to a Chinese fever, or the epidemy of Philadelphia, as that of Spain. "The next cause," says he, "is want of oxygen in the constitution." The reader would imagine that he had overlooked the first and principal cause; but this is Dr. Domeier's façon de parler. In the next paragraph, he states the remote cause to be a deficiency of oxygen in the atmosphere. From a philosophic physician, as the author declares himself to be, we should have expected a detail of eudiometrical experiments, to ascertain the purity of the atmosphere, before he confidently assigned a particular deficiency in its component parts, as both the remote and proximate cause of the disease he pretends to elucidate: but Dr. D. contents himself with telling us the will-o'-the-wisps were busy that season; that hurricanes were observed in the West Indies, and volcanic eruptions in the kingdom of Naples; that therefore the atmosphere was polluted by an extraordinary quantity of carbonic gas, which diluting and diminishing the just proportion of oxygen, produced the malignant fever at Malaga and Gibraltar. Supposing there really was this amazing quantity of carbonic gas evolved in an eruption of Vesuvius, the carbonic gas, which is but a dull traveller, had three hundred and fifty miles to traverse before it could arrive to infect the atmosphere of Malaga and Gibraltar, while its own neighbourhood, which, by its specific gravity, it should first fall upon, remained free from any contagious fever.

From a train of reasoning or *arguing*, as the author more properly calls it, he declares, and hopes he convinces the reader, "that the usual absorbing powers and pressure of the atmosphere are diminished, and being in want of oxygen it absorbs this substance more eagerly from the surface of the body than usually." Now this is one of the passages which is to me unintelligible. If "the absorbing power of the atmosphere is diminished," how can it possess an unusual power of absorption? Its pressure, one one would suppose to be augmented not diminished, as carbonic gas is half again as heavy as its other component parts. The yellowness of the skin he considers as a consequence of the general cause, want of oxygen; and the practice of oiling the bodies of the sick as intended to prevent the escape of the fluid. It is the first time, I believe, that it has been understood that the bodies of healthy or sick animals gave out oxygen; this therefore is another discovery.

From this satisfactory view of the epidemic of Spain, the history of the symptoms making no part of the Essay, the author proceeds to the method of cure, and this he hopes to compose with the more facility as he has but one indication, viz. to supply the system with oxygen.— For this purpose he recommends a liberal exhibition of vitriolic acid, which he considers as the "best of remedies," and proposes a dose of from 30 to 100 drops several times a day. This is indeed bold practice. Genuine wine he recommends as the best common drink, and desires the patient to be exposed much to the air, and carried about in open carriages, waggons, chairs, and on horseback. The latter part of this advice, I imagine, the physician would seldom have an opportunity of putting in practice until the patient's fate was decided, and he pretty well advanced in his cure; beside, the exposure to this vitiated air would only facilitate that "eager absorption from the body," which he talks of, and which he says the oiling was intended to prevent; and the unlimited exhibition of genuine wine, by increasing the action of the system, would certainly cause an increased expenditure of that principle which he desires to save and supply. His other ideas on this part of the subject are not novel, and are within the comprehension of every nurse-tender.

In his "proposals to prevent the progress of an epidemic," which forms the next part of his essay, among many common-place directions, concerning cleanliness, ventilation, fresh diet, &c. I was surprised to find a recommendation to encourage public amusements and shows, as, by collecting a number of persons together in an atmosphere already unfit for the purposes of respiration, the most fatal consequences might be apprehended; nor, upon his principles, is it easy to comprehend why he recommends gymnastic exercises, the use of wine, brandy, and porter, as all those encourage the expenditure of oxygen in the human body, when the source from which it is to be supplied is supposed to be already exhausted.

 Dr.

Dr. Domeier offers an odd apology for not confining his treatise to the history of a particular epidemic, because he says, "it would lead him too far from his purpose, and for this limited space." The latter part of this apology, tho' not very clear, alludes I suppose to want of room in the Journal for a more copious treatise. But if he had any purpose in writing this essay, one would imagine from the title of it, that it was intended to apply to a particular epidemic; and a definite subject may surely be described in fewer words than an indefinite one. Of all classical quotations, affixed as mottoes to philosophical or medical essays, the most miserable is, I think, that hacknied and trite one, Si quid novisti rectius, &c. However palpable the absurdity of a theory, if the subject is of a speculative nature, or the proofs of a more rational one difficult, or impossible to be produced, the theorist triumphantly, as he imagines, fortifies his system with this quotation; yet this is an argument the Doctor uses, to support his idea of the atmosphere being loaded with carbonic acid gas.

